# Axial Micromotion Locking Plate Construct Can Promote Faster and Stronger Bone Healing in an Ovine Osteotomy Model

**DOI:** 10.3389/fbioe.2020.593448

**Published:** 2021-01-15

**Authors:** Zhihua Han, Jianhong Wu, Guoying Deng, Chun Bi, Jiandong Wang, Qiugen Wang

**Affiliations:** ^1^Trauma Center, Department of Orthopaedics and Traumatology, Shanghai General Hospital, Shanghai Jiaotong University, Shanghai, China; ^2^Sino-Euro Orthopaedics Network, Homburg, Germany

**Keywords:** bone healing, animal model, bone implant, locking plate fixation, interfragmentary motion

## Abstract

Fixing bone fractures with controlled axial interfragmentary micromotion improves bone healing; however, the optimal type of implant construct for this purpose is still lacking. The present study describes a novel axial micromotion locking plate (AMLP) construct that allows axial interfragmentary micromotion of 0.3 or 0.6 mm. We investigated whether the AMLP constructs enhance bone healing compared to an ordinary locking plate (LP) using an ovine osteotomy model. The stiffness of the constructs was tested under axial loading. We created a 3-mm osteotomy in the left hind leg tibia of sheep that was then stabilized with a 0.3- or 0.6-mm AMLP or LP construct (*n* = 6/group). Bone healing was monitored weekly by X-ray radiography starting from week 3 after surgery. At week 9, the specimens were collected and evaluated by computed tomography and torsional testing. We found that the AMLPs had a lower stiffness than the LP; in particular, the stiffness of the 0.6-mm AMLP construct was 86 and 41% lower than that of the LP construct for axial loads <200 and >200 N, respectively. In the *in vivo* experiments, tibial osteotomies treated with the 0.6-mm AMLP construct showed the earliest maximum callus formation (week 5) and the highest volume of bone callus (9.395 ± 1.561 cm^3^ at week 9). Specimens from this group also withstood a 27% greater torque until failure than those from the LP group (*P* = 0.0386), with 53% more energy required to induce failure (*P* = 0.0474). These results demonstrate that AMLP constructs promote faster and stronger bone healing than an overly rigid LP construct. Moreover, better bone healing was achieved with an axial micromotion of 0.6 mm as compared to 0.3 mm.

## Introduction

Stable fixation, preservation of the periosteal blood supply, and early functional rehabilitation are the main goals of bone fracture repair. Locking compression plates and locking screws allow stabilization of the fracture site. However, incorrect working length, plate length, and screw placement can cause overly rigid fixation, which can delay natural bone healing by suppressing callus formation at the near cortex, preventing union, or promoting nonunion, potentially leading to construct failure (Foux et al., 1997; Vallier et al., 2006; Henderson et al., 2008, 2010; Lujan et al., 2010; Bogunovic et al., 2013; Bottlang et al., 2016; Elkins et al., 2016; Hofmann-Fliri et al., 2020). The notion that less rigid fixation actually enhances fracture healing is now widely accepted. A biomechanical study in dogs demonstrated that a custom-made axially flexible plating system that allowed comparable motion in the near and far cortices improved bone healing (Foux et al., 1997; Elkins et al., 2016). Fracture healing is also enhanced by biomechanical stimulation; interfragmentary micromotion is thought to be a prerequisite for healthy fracture union (Elkins et al., 2016). Additionally, controlled axial dynamization was shown to accelerate bone healing (Kenwright et al., 1986; Gardner et al., 2010; Bottlang et al., 2016).

The locking plate (LP) system was initially designed for improved stability and reduced bone-plate compression (Bottlang et al., 2016); it also preserves the blood supply in biological bridge plating, allowing functional reduction for complex fractures (Tsai et al., 2015). LPs are amenable to modifications that mitigate stiffness while providing relatively stable fracture fixation. Construct stiffness can be varied by altering plate length, working length, screw position, and screw number (Ellis et al., 2001; ElMaraghy et al., 2001). However, no specific guidelines have been established on how to determine these parameters in individual cases to optimize outcome.

Many construct modification strategies have been developed to reduce the stiffness of LPs including dynamic locking screws (Pohlemann et al., 2015), near cortical slots (Gardner et al., 2010) and far cortex locking screws (Bottlang et al., 2010), near cortical dynamic locking screws (Richter et al., 2015), and biphasic plating (Hofmann-Fliri et al., 2020). For example, an active LP with sliding elements embedded in a silicone envelope enabled axial interfragmentary motion up to 1.5 mm (Madey et al., 2017). In an ovine model, tibial osteotomies treated with active LPs had more callus at follow-up and were considerably stronger than those treated with LPs. The active LP has also been tested in a prospective study involving 11 humeral shaft fracture cases (Madey et al., 2017). However, active LPs have a complex design and are difficult to manufacture.

Based on this concept, we developed a new axial micromotion (AM) LP construct that enables controlled axial interfragmentary motion but with simpler design. In the present study, we evaluated the AMLP construct in terms of stiffness and effect on bone fracture healing as determined by callus formation and bone strength in an ovine transverse osteotomy model.

## Materials and Methods

### Study Design

Animals were purchased and used under the approval of the animal care committee of Shanghai General Hospital, Shanghai Jiaotong University, Shanghai, China. We used an established and commonly used ovine tibia osteotomy and fracture healing model in this study. Briefly, a 3-mm full-thickness osteotomy was created in the middle portion of the tibia of the left hind leg, and the implant construct was applied. To facilitate the comparison of results, the study protocol and sample size were consistent with previous studies investigating LP dynamization (Bottlang et al., 2016). The sheep (*N* = 18) were randomly assigned to receive one of three different types of construct: ordinary LP (LP group, *n* = 6), AMLP with 0.3-mm axial micromotion (0.3-mm AMLP group, *n* = 6), or AMLP with 0.6-mm axial micromotion (0.6-mm AMLP group, *n* = 6). Bone healing was monitored weekly starting from 3 weeks after surgery by X-ray radiography. All animals were euthanatized at postoperative week 9, and callus volume was evaluated by quantitative computed tomography (CT), while the mechanical strength of healed bone was tested by applying torsion until failure.

### LP Constructs

**Figure 1:**
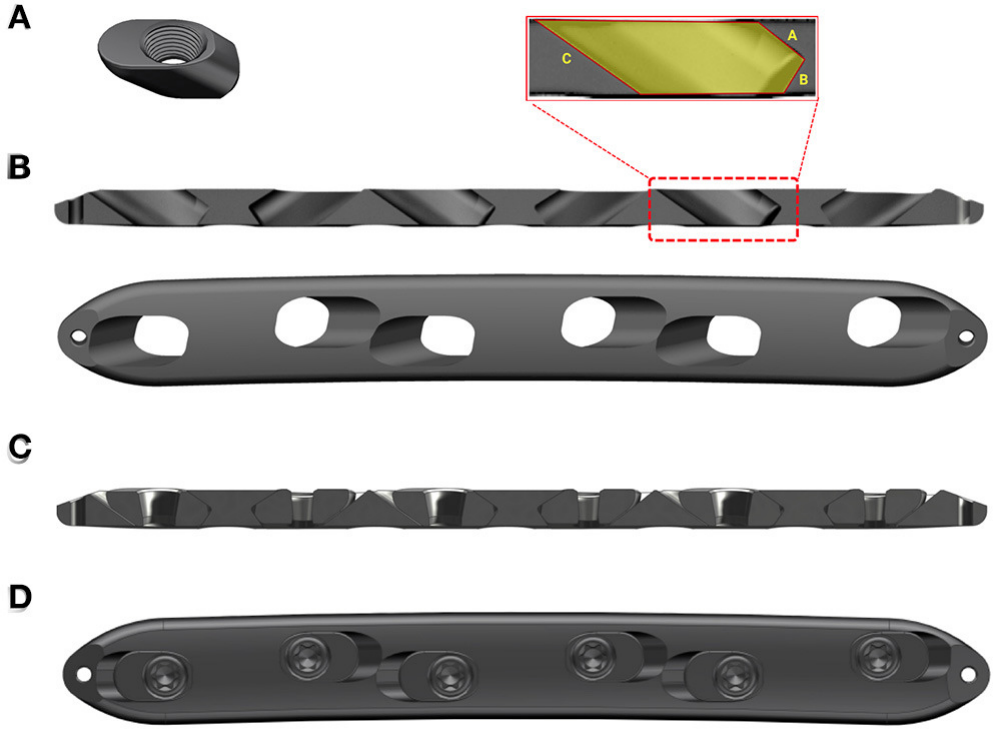
Axial micromotion locking plate (AMLP) construct. **(A)** Sliding wedge with internal thread. **(B)** Cross-sectional and superior views. **(C)** Cross-sectional view of the AMLP construct without screws. **(D)** Superior view of the whole AMLP construct.

All constructs in this study were made of the same material (titanium alloy; Ti−6AI−4V, TAV) (Figure 1). All LPs were typical 4.5-mm large fragment plates with a length of 130 mm, width of 15 mm, and thickness of 5 mm. The plates had six locking holes in a staggered configuration. For the AMLP constructs, micromotion was enabled by a threaded gliding wedge (Figure 1A) integrated at each slot that was sculpted in a pentagon shape from the cross-sectional view (Figure 1B). Facet *A* prevents the screw from being pulled out. Also together with facet *B*, they not only control the motion of the sliding wedge at the theoretical range in both directions but also function in axial load bearing. Facet *C* allows the backward motion of the sliding wedge and also play roles in force transduction. The micromotion was maintained by the difference in size between the sliding wedge and slot. Importantly, the slots were oppositely placed to ensure that the micromotion was controlled in both directions (Figure 1C). All implants used in the study were prototype constructs designed for the ovine 3-mm osteotomy model and were manufactured by BETK Corp. (Jiangsu, China).

### Biomechanical Assessment of AMLP Constructs

Stiffness under axial loading was evaluated for all three constructs by bench testing three plates per group. The constructs were used to bridge 3-mm gap osteotomies in cylindrical bone surrogates (diameter, 27 mm and wall thickness, 7 mm; Sawbones, Vashon, WA, USA; #3403-10). All tests were performed with a materials testing system (CMT6104; MTS Systems, Shenzhen, China). Axial quasi-static ramped load was applied up to 1,000 N at a rate of 50 N/s, and the load–displacement curve was recorded using a dedicated workstation.

### Animal Surgery

Sexually mature male China-Hu sheep (*N* = 18) with a mean weight of 45 kg and age of 8 months were used in the animal experiments. The ovine tibia osteotomy model was established as previously reported (Bottlang et al., 2010, 2016). The surgical procedure is illustrated in Supplementary File 2. Briefly, under general anesthesia and after administration of antibiotics and analgesics, a 13-cm-long skin incision was made over the medial tibia of the left hind leg, and the tibial shaft was exposed by displacing the subcutaneous tissue. The custom template was slightly contoured to fit the tibial shaft and affixed to both ends of the bone using Kirschner wires. Six holes were drilled according to the template using a 4.0-mm drill bit. After fastening the template to the bone with six 4.5-mm cortical screws, the osteotomy was created using an oscillating saw blade through the guiding slot under constant irrigation. The osteotomy size was restricted to 3 mm because the distance between guiding holes in the drill template was 3.0 mm shorter than that in the plates. The osteotomy was stabilized with either an AMLP or LP construct along with 4.5-mm bicortical locking screws. Wound closure was performed layer by layer. A cylindrical cast was applied to the injured limb for the first 3 weeks after the operation to prevent tibial fracture secondary to bending loads while allowing axial loading and walking immediately after surgery (Bottlang et al., 2010, 2016; Richter et al., 2015). Antibiotics (benzylpenicillin and gentamicin) and analgesics (carprofen and buprenorphine) were administrated in the first 3 days after surgery (Bottlang et al., 2016). Animals were individually housed in a 4-m^2^ pen for the first 2 postoperative weeks before being transferred to a 20-m^2^ six-sheep pen until the time of sacrifice (postoperative week 9).

### Radiographic Evaluation

Serial X-ray examinations were performed immediately after the surgery and then weekly from postoperative weeks 3 through 9. Radiographs of lateral and anterior–posterior views of the osteotomy were archived; the cast was removed before radiography to eliminate interference and then reapplied to the animal afterward. Radiographs were blindly and independently assessed in terms of projected callus area (PCA) of the periosteal callus at the anterior–posterior and lateral aspects (Supplementary File 1) using a software program by two independent senior radiologists according to a previously reported protocol (Bottlang et al., 2010).

### CT Examination

All osteotomy specimens were harvested at week 9, and the implant removed and examined by CT (Model iCT 256; Philips, Farmington Hills, MI, USA). The parameters of the CT scan were as follows: kilovoltage peak = 120, slice thickness = 0.8 mm, and space between slices = μm; the protocol name was Knee/Orthoped. The three-dimensional total bone volume was obtained for each excised tibia, and cross-sectional area at the osteotomy site in relation to the amount of newly formed bone was measured. Callus volume was automatically rendered by differentiating soft tissue from callus and callus from cortical bone with consistent thresholds of 600 and 1,600 HU, respectively.

### Biomechanical Analysis of Cadaveric Specimens

Biomechanical tests of tibia specimens were performed with a material testing system (Tabletop axial torsion load frames, Model306; Shore Western, Monrovia, CA, USA). Biomechanical properties of healing including stiffness, strength, and energy to failure were evaluated and compared after being normalized to the contralateral healthy tibiae. Tibia specimens were harvested at postoperative week 9, and both ends were embedded in cement (polymethylmethacrylate) fixtures, leaving an exposed section of 180 mm; the specimens were subjected to torsion aligned with the tibial shaft axis. A rotational force of 10°/min was applied until the specimen fractured or shear movement occurred at the osteotomy gap. Torsional stiffness was determined from the linear slope of the torsional moment vs. rotation curve before it reached the highest torsional moment, which represented the torsional strength. Energy to failure was calculated by integrating the area under the torsion vs. rotation curve up to the peak torsional moment at which fracture occurred (Bottlang et al., 2010).

### Statistical Analysis

Data are presented as mean ± standard deviation. Statistical analyses were performed using Prism 8.0 software (GraphPad, La Jolla, CA, USA). Data normality was verified with the D'Agostino and Pearson omnibus normality test. Statistical differences between groups were evaluated by one-way analysis of variance. *P* < 0.05 was considered statistically significant.

## Results

All animals (*N* = 18) underwent the experimental procedure without complications or postoperative surgical infection. Weight-bearing activity was allowed for all sheep starting from the first day after surgery, and no events were observed until the animals were sacrificed at week 9.

### Construct Stiffness

**Figure 2:**
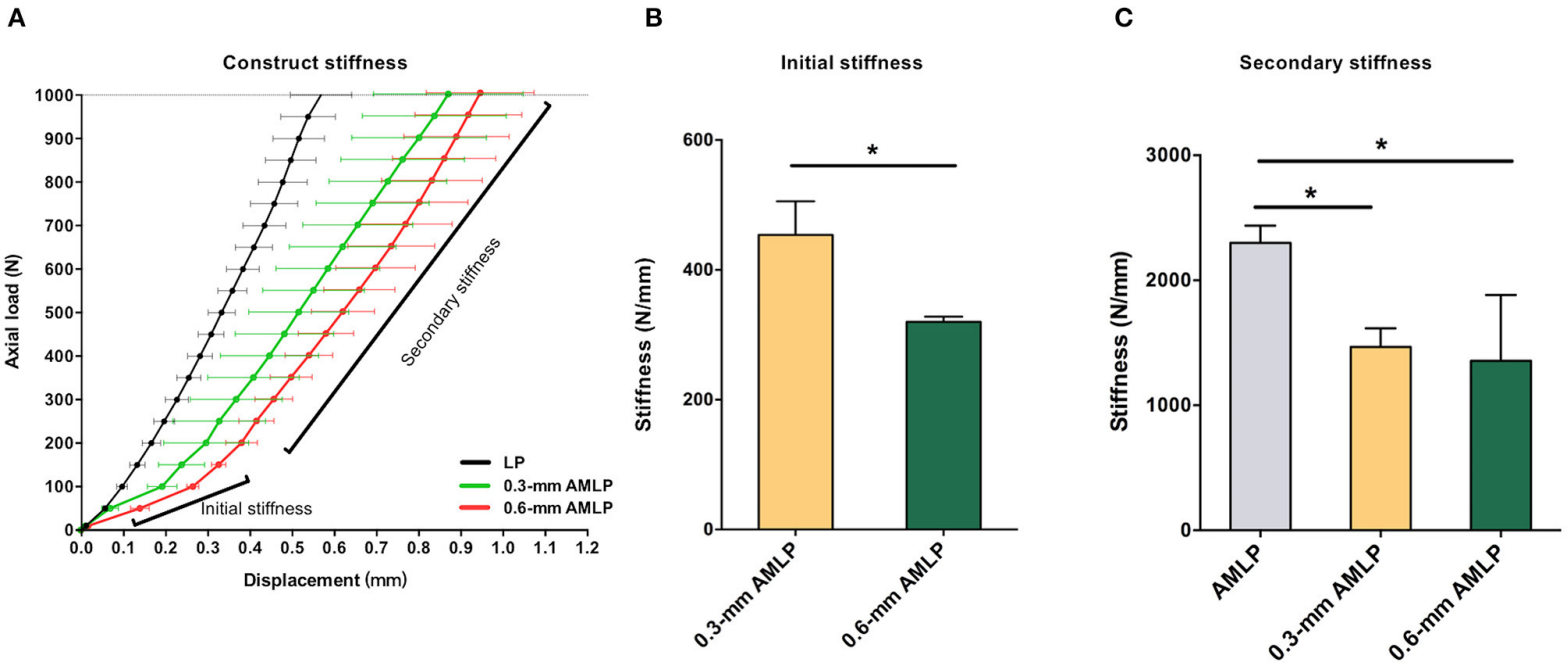
Stiffness of 0.3 and 0.6 mm AMLP constructs and LP construct. **(A)** Initial stiffness of the 0.6-mm AMLP construct was 86 and 30% lower than that of the LP and 0.3 mm AMLP constructs, respectively, and **(B)** the 0.6 mm AMLP possessed significantly lower initial stiffness compared to 0.3 mm AMLP. **(C)** Both types of AMLP construct had lower secondary stiffness than the LP construct. *P < 0.05.

Mechanical testing confirmed the lower stiffness of the AMLP constructs compared to the LP constructs. The latter showed stiffness at 2,299 ± 138.2 N/mm. AMLP constructs displayed their initial stiffness for the load below 200 N. The initial stiffness of the 0.6-mm AMLP constructs was 86% lower than that of the LP construct (Figure 2A), while such value was 80% for the 0.3-mm AMLP constructs. The initial stiffness of the 0.3-mm AMLP construct was 29.5% higher than that of the 0.6-mm construct (453.7 ± 51.90 vs. 319.8 ± 8.047 N/mm; Figure 2B). The secondary stiffness of AMLP constructs was demonstrated at axial loads exceeding 200 N (Figure 2C) and was similar between the two AMLP constructs (0.3 mm: 1,467 ± 148.5 N/mm; 0.6 mm: 1,356 ± 526.4 N/mm). These values were 36 and 41% lower, respectively, than the stiffness of the LP construct (Figure 2C).

### Radiographic Assessment of Callus Formation

**Figure 3:**
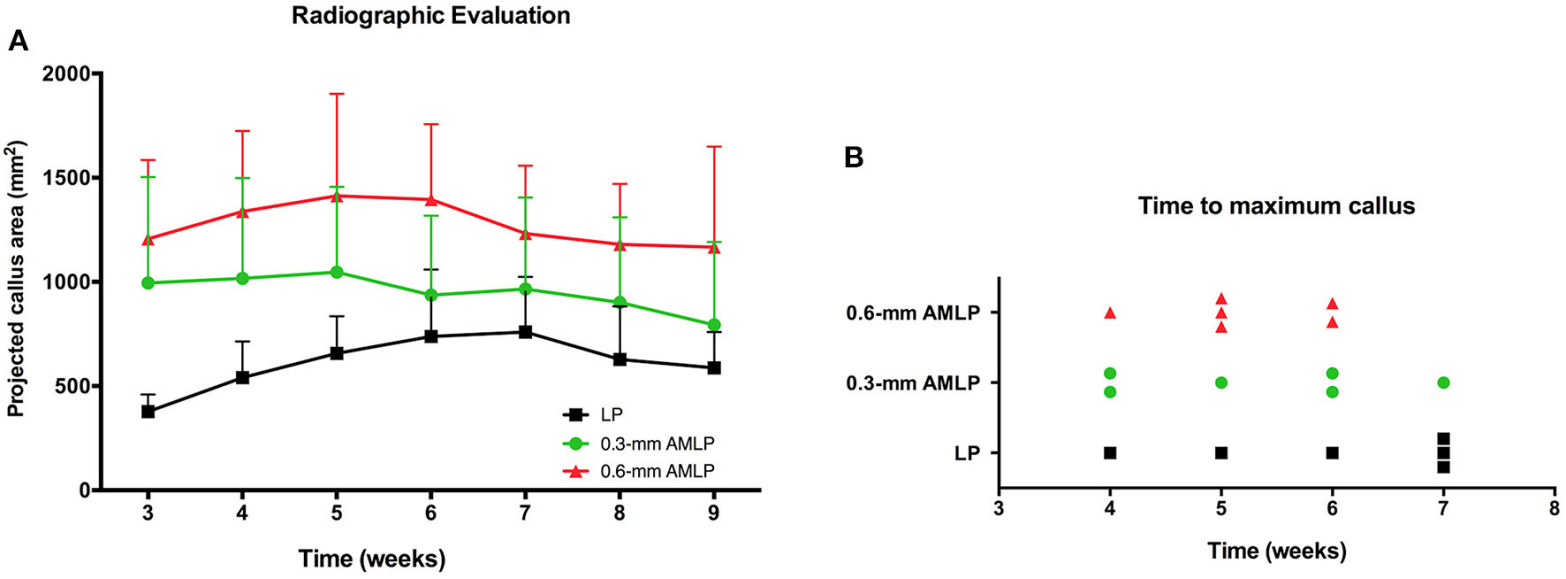
Periosteal callus formation and callus area at postoperative weeks 3–9. **(A)** The 0.6-mm axial micromotion locking plate (AMLP) group had the highest PCA throughout the observation period and had a larger PCA than the LP group at each time point (all *P* < 0.05). **(B)** Tibial osteotomies treated with 0.6-mm AMLP construct achieved maximum callus formation earlier than the other groups.

**Figure 4:**
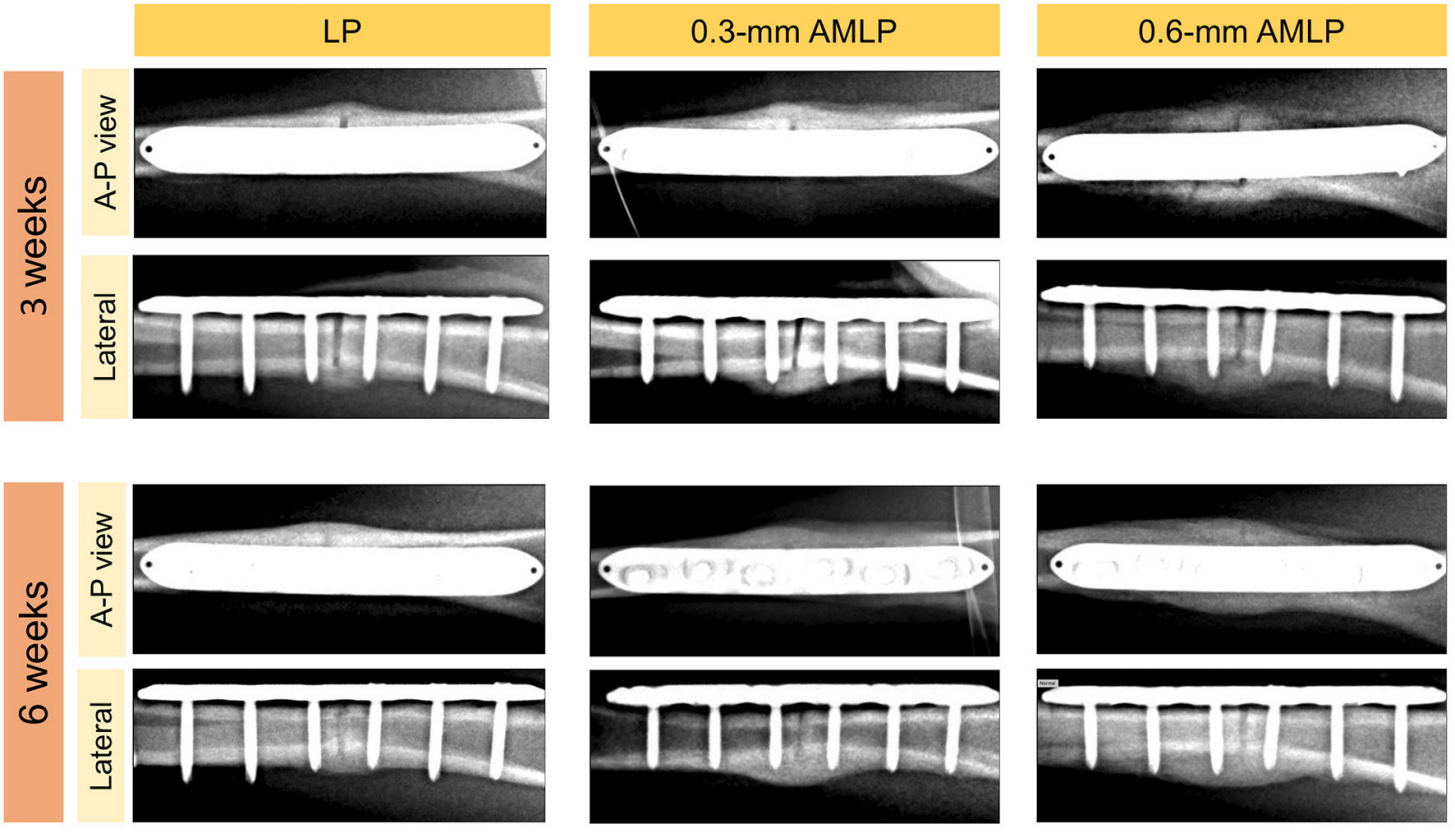
Representative X-ray images at postoperative weeks 3 and 6. The periosteal callus formed more symmetrically in the 0.6-mm axial micromotion locking plate (AMLP) group in the A-P view; in the lateral view; more periosteal callus formed to bridge the osteotomy gaps in the AMLP groups than in the LP group.

Weekly radiographic monitoring revealed the largest PCA in the 0.6-mm AMLP group (Figure 3), which was larger than that of the LP group at each time point (*P* < 0.05; Table 1). More specifically, the 0.6-mm AMLP group had a PCA of 1,207 ± 378.4 mm^2^ at postoperative week 3—which was much larger than the other two groups—and reached a maximum value of 1,413 ± 490.0 mm^2^ at postoperative week 5 (Figure 3A). Similarly, the maximum PCA for the 0.3-mm AMLP group (1,046 ± 409.4 mm^2^) was observed at postoperative week 5. The LP group not only had the smallest PCA among the three groups, but the maximum PCA was 2 weeks later (postoperative week 7) than for the AMLP groups (Figure 3A). At postoperative week 6, all tibial specimens (6/6) in the 0.6-mm AMLP group and 5/6 in the 0.3-mm AMLP group had reached the maximum PCA, as compared to 3/6 specimens in the LP group (Figure 3B). Periosteal callus formation in the 0.6-mm AMLP group progressed more symmetrically at both anterior and posterior aspects of the osteotomy compared with the LP group in the anterior–posterior view of the radiograph, whereas in the lateral view, the osteotomy had a larger PCA bridging the osteotomy gap (Figure 4).

**Table 1:** Chronologic changes in the projected callus area of three groups.

**Time**	**Group**		
	**LP**	**0.3-mm AMLP**	**0.6-mm AMLP**
Week 3	377.4 ± 81.51	994.5 ± 507.9^*^	1,207 ± 378.4^**^
Week 4	540.0 ± 173.9	1,017 ± 481.1^*^	1,338 ± 385.9^**^
Week 5	657.1 ± 177.9	1,046 ± 409.4	1,413 ± 490.0^*^
Week 6	738.1 ± 321.3	936.2 ± 381.5	1,395 ± 361.9^*^
Week 7	759.3 ± 264.1	965.3 ± 439.3	1,232 ± 326.1^*^
Week 8	628.1 ± 254.7	901.4 ± 407.9	1,180 ± 289.7^*^
Week 9	587.4 ± 171.6	793.7 ± 397.6	1,167 ± 482.6^*^

* *P < 0.05*,

** *P < 0.01 vs. LP group*.

### CT Evaluation

**Figure 5:**
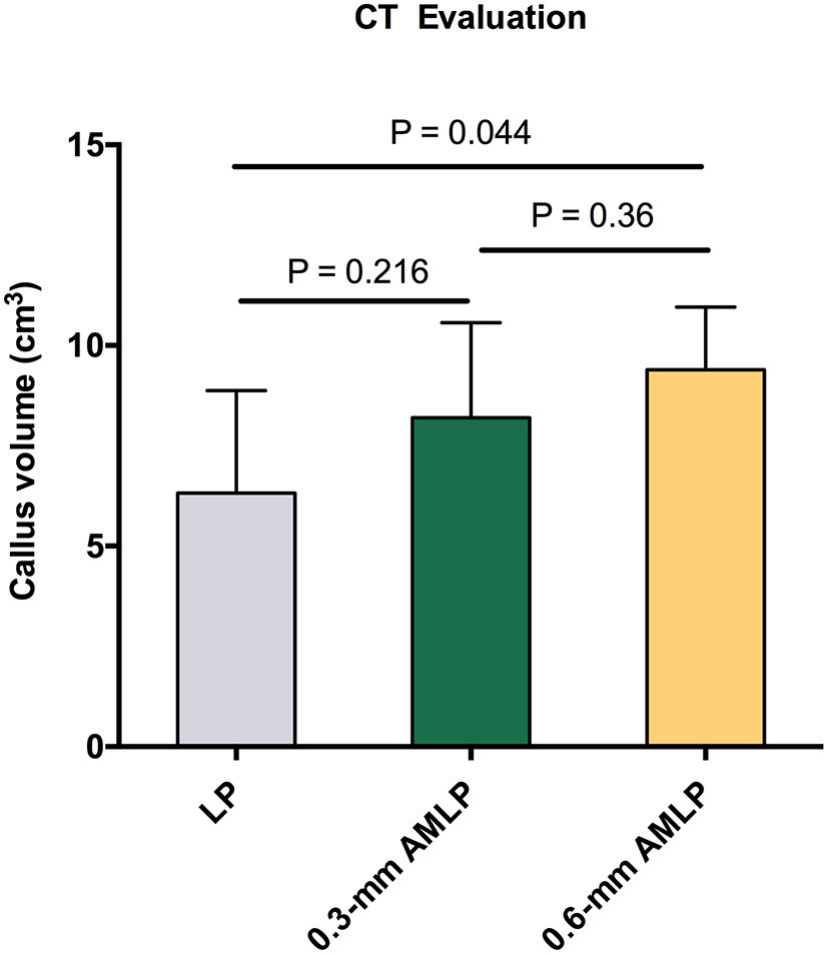
Total callus volume assessed by CT. The 0.6-mm axial micromotion locking plate (AMLP) group had the most callus at postoperative week 9 (9.395 ± 1.561 cm^3^), with a statistically significant difference relative to the locking plate (LP) construct (*P* = 0.044).

**Figure 6:**
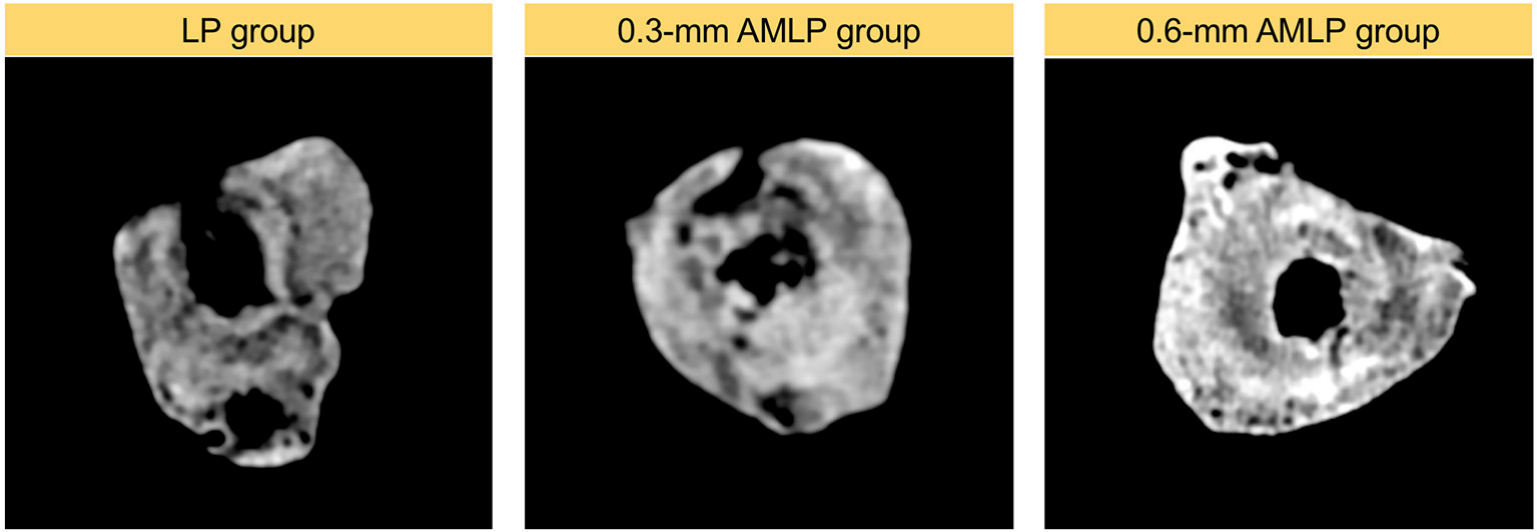
Cross-sectional area at the osteotomy gap. Callus formation in the osteotomy gap of the LP group was asymmetric. The callus extended to all cortical areas in both axial micromotion locking plate (AMLP) groups and homogeneously filled the osteotomy gap in the 0.6-mm AMLP group.

Total callus volume measured by CT at postoperative week 9 was larger in the 0.6-mm AMLP group (9.395 ± 1.561 cm^3^) than in the 0.3-mm AMLP group (8.198 ± 2.369 cm^3^; *P* = 0.36) and significantly greater than that in the LP group (6.317 ± 2.558 cm^3^; *P* = 0.044) (Figure 5). Transverse sections through the unhealed osteotomy gap (Figure 6) revealed that the osteotomy treated with 0.6-mm AMLP developed a circumferential callus around the entire cortex, especially beneath the plate. A similar but less homogeneous callus was seen in the 0.3-mm AMLP group.

### Mechanical Testing

**Figure 7:**
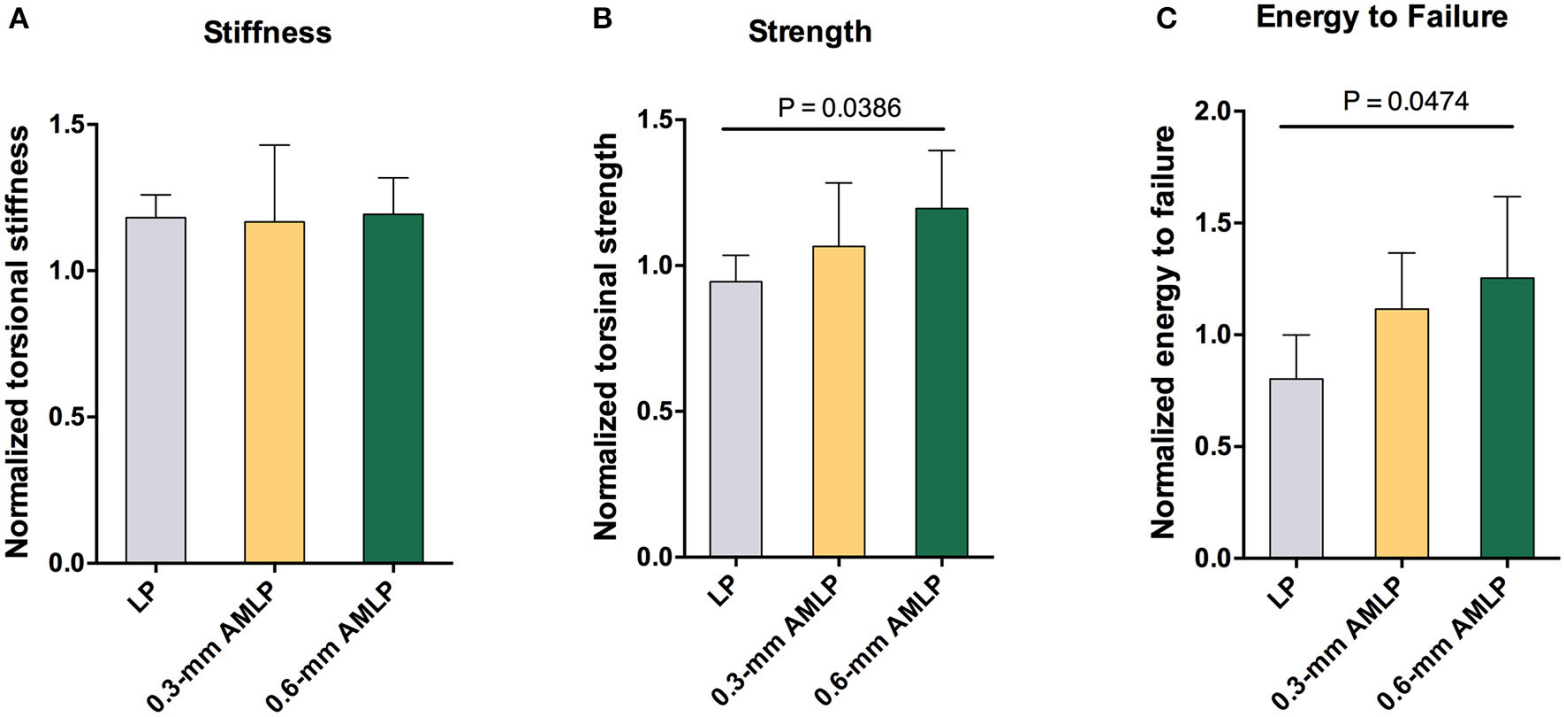
Assessment of torsion after construct removal at postoperative week 9. **(A)** Tibia in the 0.6 mm axial micromotion locking plate (AMLP) group had 1.193-fold stiffness as the native strength (1.193 ± 0.1247), similar to those in two other groups (0.3 mm AMLP: 1.181 ± 0.0777; and LP: 1.167 ± 0.0263). Osteotomies treated with AMLP constructs had the **(B)** highest maximum strength and **(C)** energy to failure.

After implant removal, the torsional stiffness evaluation showed no significant difference among all three groups: 1.193 ± 0.1247 for the 0.6-mm AMLP group, 1.181 ± 0.0777 for the 0.3-mm AMLP group, and 1.167 ± 0.0263 for the LP group. However, the 0.6-mm AMLP group withstood a 27% greater torque before failure (Figure 7) than the LP group (1.196 ± 0.1990 vs. 0.9447 ± 0.0905, *P* = 0.0386). The strength of the 0.6-mm AMLP specimens, expressed in terms of energy required to induce failure was 53% greater than in LP specimens (*P* < 0.0474).

## Discussion

In the present study, we demonstrated that an LP with controlled axial micromotion promotes faster and stronger bone healing in an ovine osteotomy model, compared with ordinary LP, with better bone healing with interfragmentary axial motion at 0.6 mm than at 0.3 mm. These results are in line with previous work demonstrating that LP constructs with reduced stiffness achieved better outcomes for bone healing (Gardner et al., 2010; Bottlang et al., 2016; Mitchell, 2016).

Callus formation occurred more rapidly in osteotomies treated with AMLP constructs compared to the LP construct (Figure 3). Likewise, Bottlang et al. found that the osteotomies treated with “active locking plate” had significantly higher amount of callus than those treated with locking plates at all time points; also the peak of periosteal callus appeared earlier in the “active locking plate” treated group (Bottlang et al., 2016). They found similar results in the study about “far cortical locking” (Bottlang et al., 2010). Importantly, in the present study, the callus was formed in a circumferential pattern in the 0.6-mm AMLP group but not in the other two groups (Figure 4). Thus, the AMLP constructs provided symmetric axial motion, although 0.3 mm was insufficient for the fracture to achieve comparable motion at the near cortex.

Both AMLP groups (0.3- and 0.6-mm AMLP) showed enhanced callus formation relative to the LP group, as evidenced by the larger peak PCAs in the weekly radiographs and higher total callus volumes in the CT scan. The larger amount of periosteal callus formed by relatively stable fixation could promote early functional recovery and more stable healing. In the torsional test, specimens in the 0.6-mm AMLP group had higher torsional strength (Figure 7B) and absorbed 53% more energy until failure (Figure 7C). Greater healing strength is clinically important to ensure weight-bearing rehabilitation and shorten the load-sharing duration of the fixation construct.

Our findings are, to some extent, in line with previous reports. Bottlang et al. demonstrated that 3-mm ovine tibial osteotomies treated with an active LP construct with 1.5-mm axial motion had approximately six times more periosteal callus at week 3 and had greater torsional resistance at week 9 than those treated with an LP construct. The benefit yielded by the active LP construct used in their preclinical work was greater than that observed in our study: specifically, there was nearly six times more callus in the active LP group, whereas in our study, the amount was three times greater with the AMLP constructs than with the LP construct. This discrepancy can be explained as follows: (1) different protocols for radiographic measurement (Supplementary File 1) used in the two studies; (2) the dynamization provided by the elastic suspension of locking holes of their active LP; and (3) the larger range of axial motion of their active LP construct than our AMLP constructs (0.3 and 0.6 mm, respectively). The latter may also contribute to the different torsional outcomes that we measured: the energy to failure was 53% greater in the 0.6-mm AMLP group than in the LP group, whereas in the study by Bottlang et al., 399% more energy was required to induce failure in specimens treated with the active LP construct. Nonetheless, our 0.6-mm AMLP construct was superior to the LP construct in terms of both callus formation and torsional strength.

The optimal interfragmentary movement at the fracture site is 0.2–1.0 mm (Claes et al., 1998; Klein et al., 2003). Our AMLP construct achieved axial motion at 0.3 or 0.6 mm, which is favorable for callus consolidation and bone formation (Mitchell, 2016). The 0.6-mm AMLP achieved better results than the 0.3-mm construct; the larger amount of callus formed with the former indicated superior biomechanical properties. However, the controlled axial interfragmentary motion of either range should improve bone healing, along with the dynamization of fracture fixation.

A semirigid implant has reduced stiffness and provides relative rather than absolute stability. Screw type and placement, bridge length, and plate type can be varied to alter stiffness; however, for these, surgeons must rely on their experience, which can lead to inconsistent outcomes. Constructs with controlled motion including the dynamic locking screw (DLS) (Röderer et al., 2014), far cortical locking screw (FLS) (Doornink et al., 2011), and active LP (Bottlang et al., 2016; Henschel et al., 2017; Madey et al., 2017 offer a more reliable solution. The DLS allows micromotion at the near cortex up to 0.45 mm via a pin-sleeve design; the FLS provides a motion envelope at the near cortex up to 0.75 mm (half of the 4.5-mm cortical tread at the far cortex minus a 3-mm bypass at the near cortex); and the active LP enables a maximum axial micromotion of 1.5 mm. Although the extent of micromotion varies, all of these constructs significantly improved bone healing in the 3-mm ovine tibia osteotomy model that was also used in our study. In contrast, the LP construct in all studies including ours resulted in defective and asymmetric callus formation.

In theory, Bottlang's active LP and our AMLP have advantages over DLS and FLS because modification at the plate vs. at the screws increases the range of micromotion, and engagement between the near cortex and FLS is replaced by engagement between the sliding element and plate, which reduces the risk of near cortex subsidence in osteoporotic cases. Moreover, our AMLP has a much simpler manufacturing process, which will substantially lower production costs and facilitate widespread bench-to-bedside application.

The present study had several limitations. First, although the ovine osteotomy model is the most widely used large animal model for bone healing investigations, it is not fully comparable to fracture healing in humans, which is a complex process influenced by many factors such as blood supply, diabetes, bone density, etc. Clinical studies are required to determine the efficacy of the AMLP construct in humans. Moreover, the maximum micromotion of our AMLP was 0.6 mm; a larger range of motion will be evaluated in future work. The control group in our study does not fully represent clinical cases, as the working length is normally extended to reduce stiffness; thus, caution must be exercised in translating the present results to clinical practice. We also macroscopically evaluated the constructs after their removal from tibial specimens, and while there was no evidence of metal debris (Supplementary File 4), this should be quantitatively evaluated to confirm the safety of the AMLP construct. The animals used in the present study were younger than the age of skeletal maturity, despite no epiphyseal line could be observed. Although three groups of the present study were mutually comparable, it still requires prudence when comparing our work with other work. Finally, the sample size was small (six animals per group), and therefore, the experiments should be repeated in larger samples to validate the results; nonetheless, we observed statistically significant differences between the AMLPs and LP constructs.

In conclusion, our study provides evidence that dynamization of fracture fixation enhances bone healing. By allowing axial interfragmentary motion, the AMLP yielded faster and symmetric callus formation and showed greater strength than overly rigid LP constructs. Additionally, a better outcome was achieved with a micromotion of 0.6 mm as compared to 0.3 mm, indicating that callus formation is partly dependent on the range of micromotion, although additional research is required to optimize the maximum motion range. Finally, multicenter clinical trials are needed to comprehensively evaluate the utility of AMLPs in treating bone fractures.

## Data Availability Statement

The raw data supporting the conclusions of this article will be made available by the authors, without undue reservation.

## Ethics Statement

The animal study was reviewed and approved by Shanghai General Hospital, Shanghai Jiaotong University.

## Author Contributions

ZH, JWu, JWa, and QW conceived and designed the study. ZH, JWu, GD, CB, JWa, and QW performed the experiments. ZH, JWa, and GD analyzed the data. ZH, JWu, and CB prepared figures and contributed to the drafting of the manuscript. QW supervised this work and edited and revised manuscript. All authors contributed to the article and approved the submitted version.

## Conflict of Interest

The authors declare that the research was conducted in the absence of any commercial or financial relationships that could be construed as a potential conflict of interest.
