# Supplementary material for: Axial Micromotion Locking Plate Construct Can Promote Faster and Stronger Bone Healing in an Ovine Osteotomy Model
Source: Front Bioeng Biotechnol. 2021 Jan 15;8:593448. doi: 10.3389/fbioe.2020.593448 (PMC7845656; doi:10.3389/fbioe.2020.593448)

Osteotomy Procedure

Step 1: place the attach the drill template onto the bone

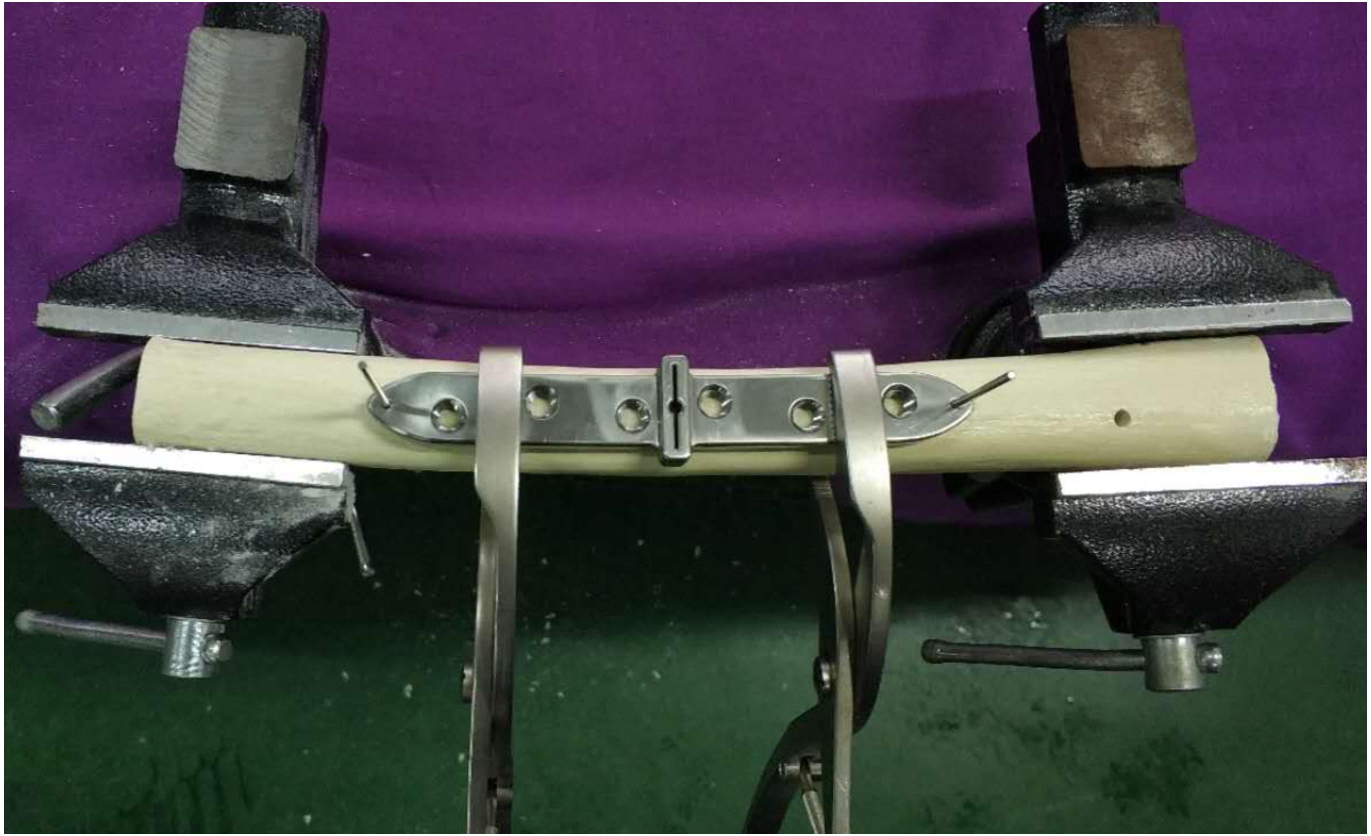

Step 2: drill two holes (3rd and 4th from left to right) adjunct to the cutting slot using drill guide

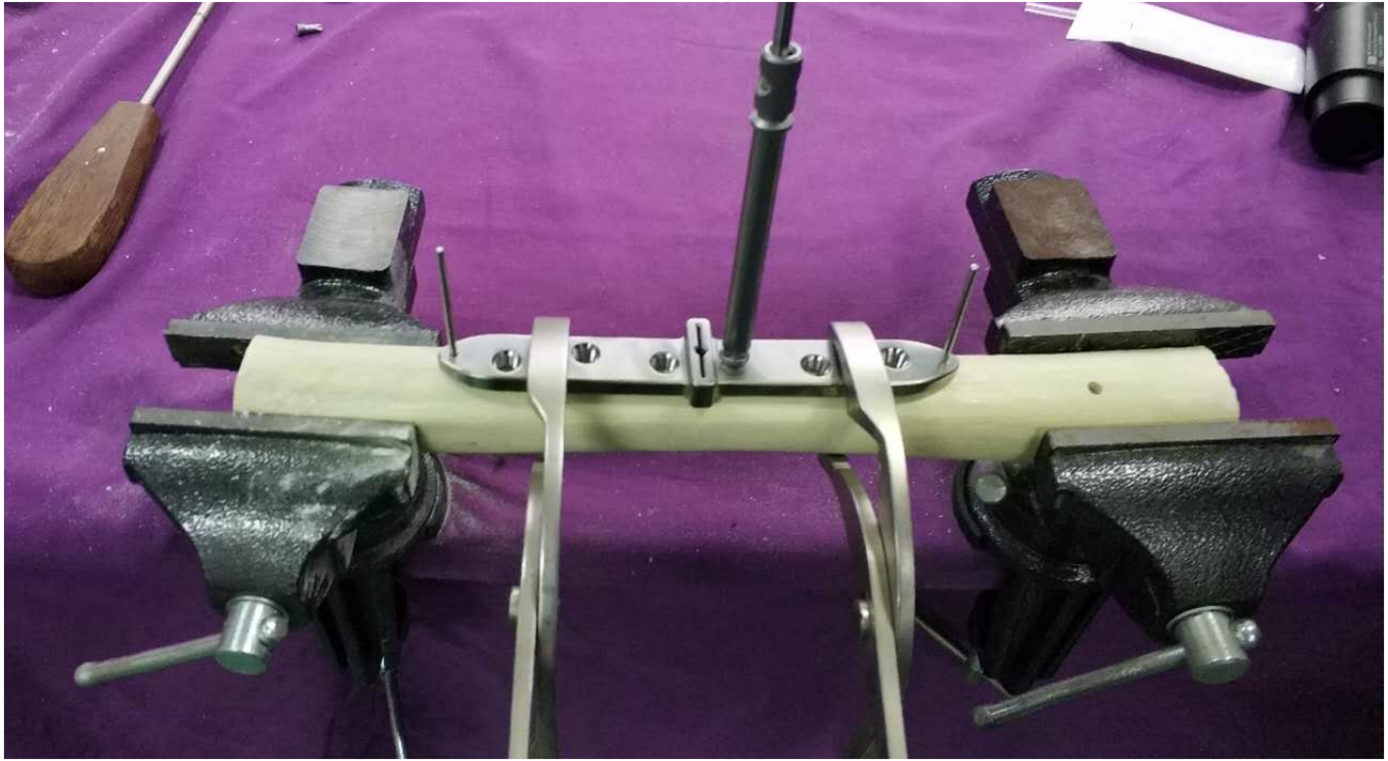

Step 3. put in 4.0-mm locking screws (14-mm length)

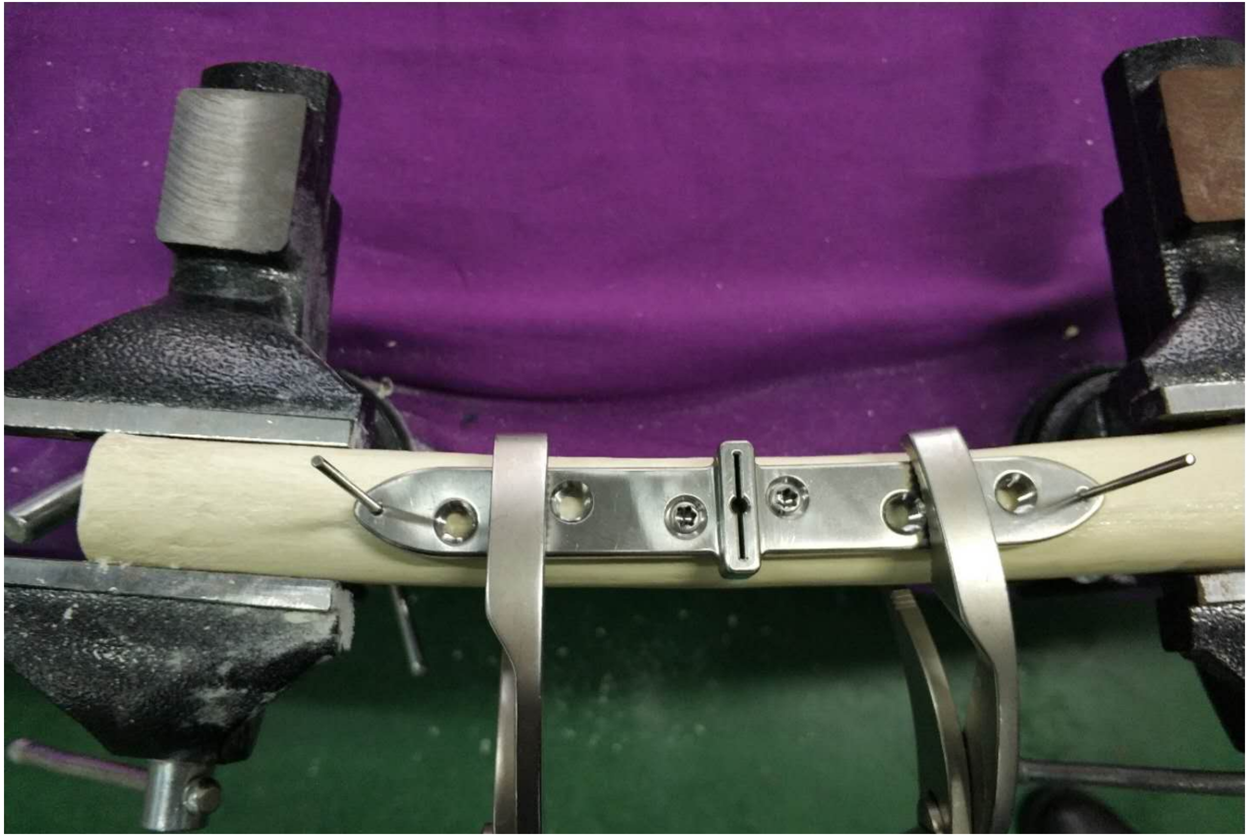

Step 4: drill other four holes and apply locking screws for temporary fixation

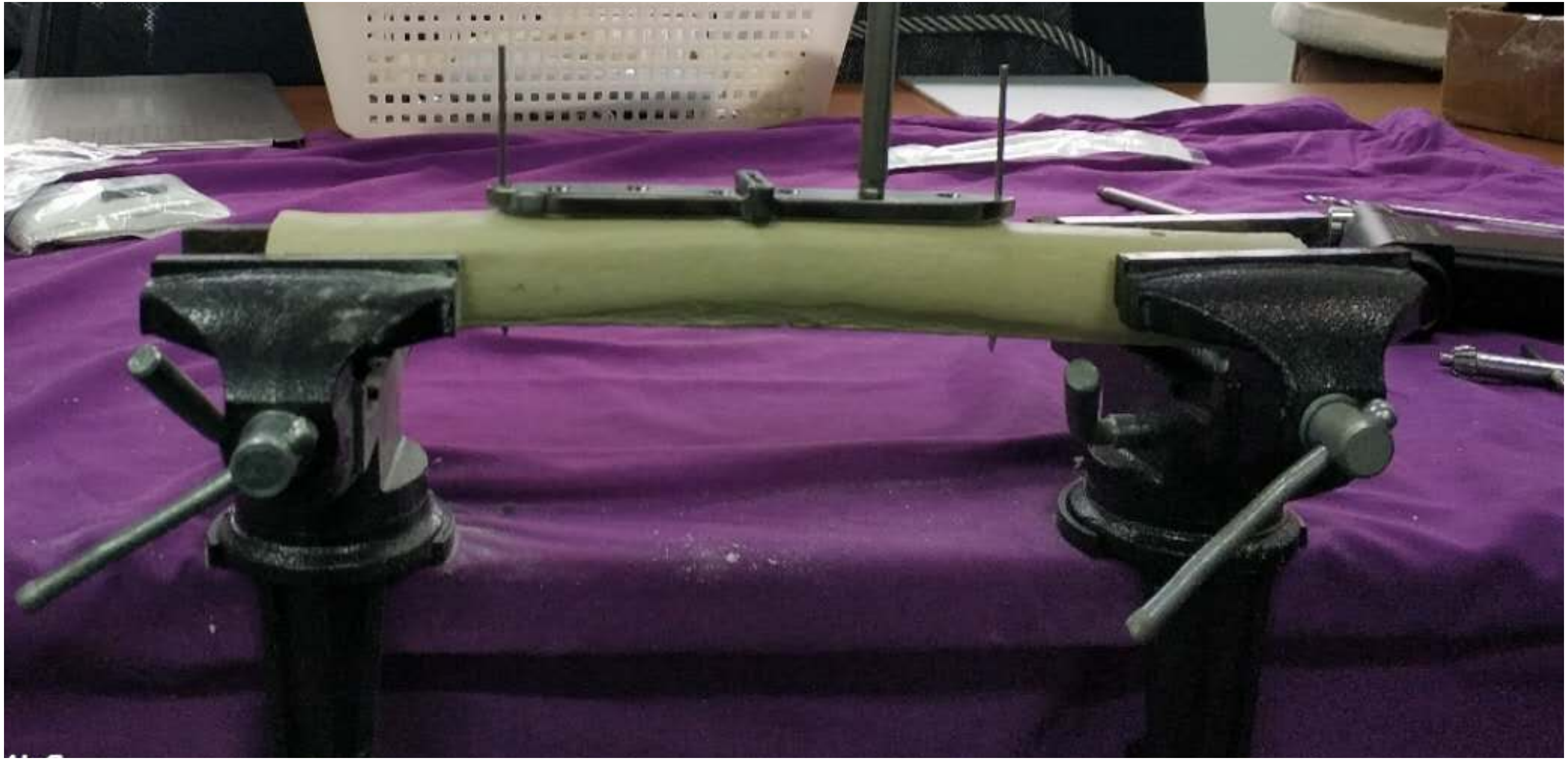

Step 5. Bicortical cutting through the cutting slot

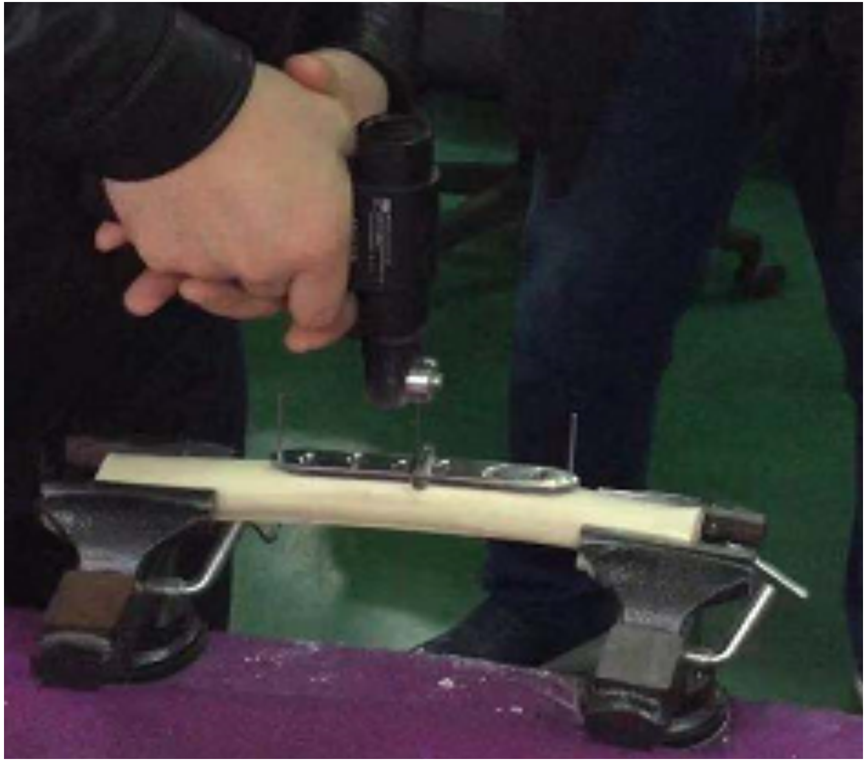

Step 6: remove the drill template and screws except the Kirsch wires

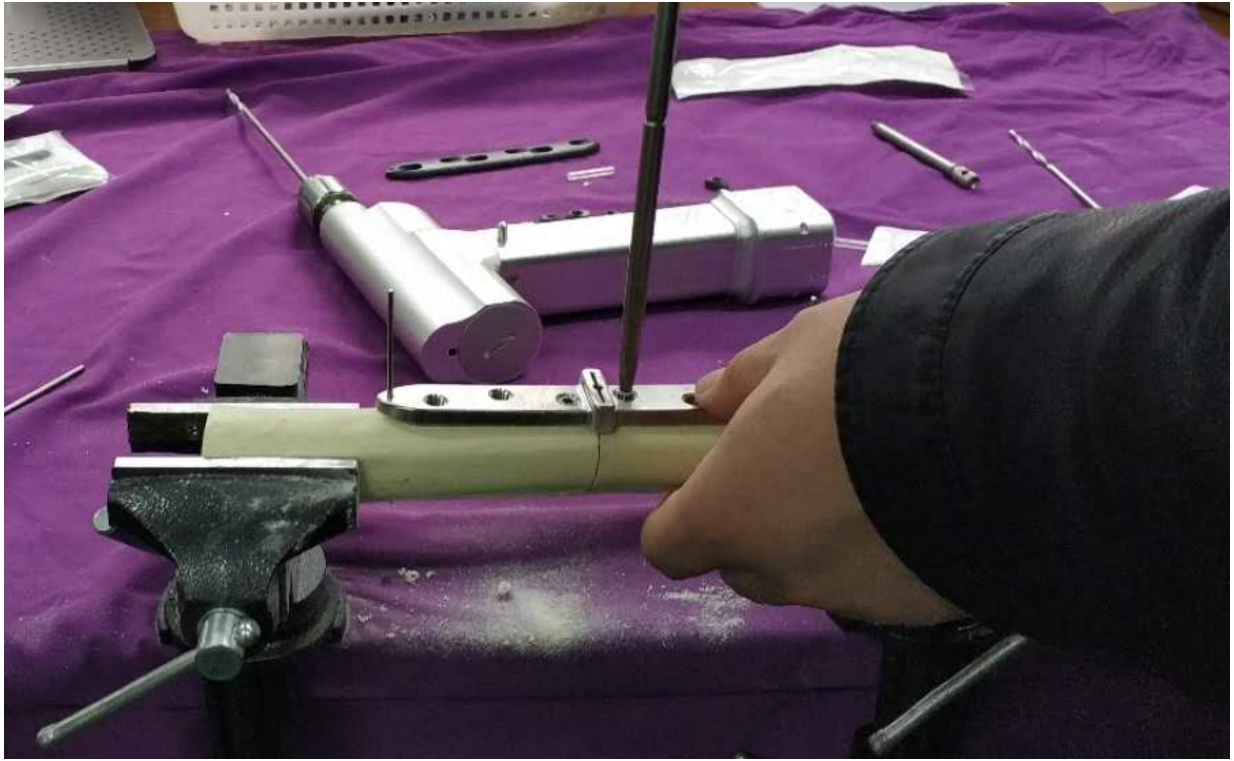

Step 7: place the dynamic locking plate according to the Kirsch wires, the osteotomy gap appears

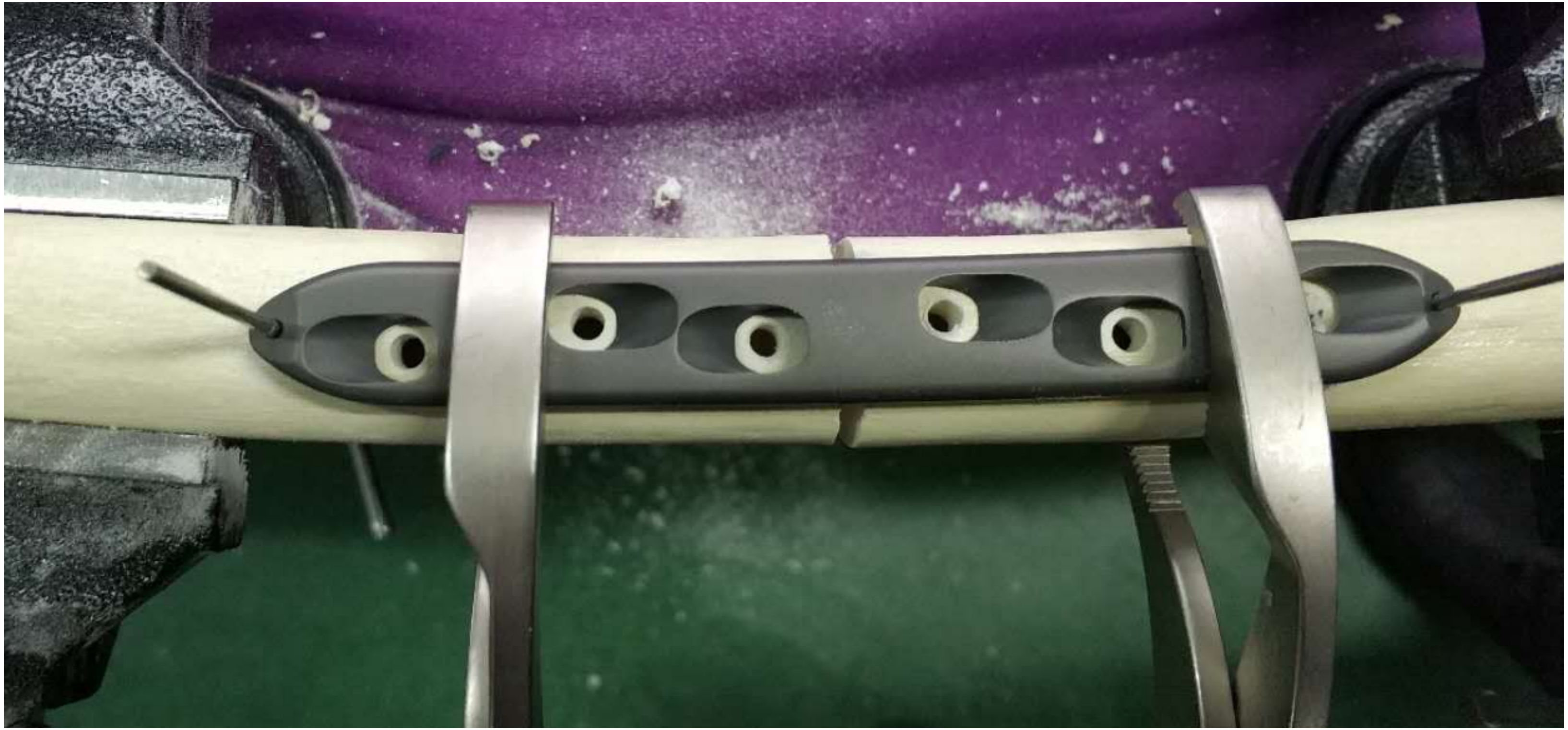

Step 8: place the sliding element

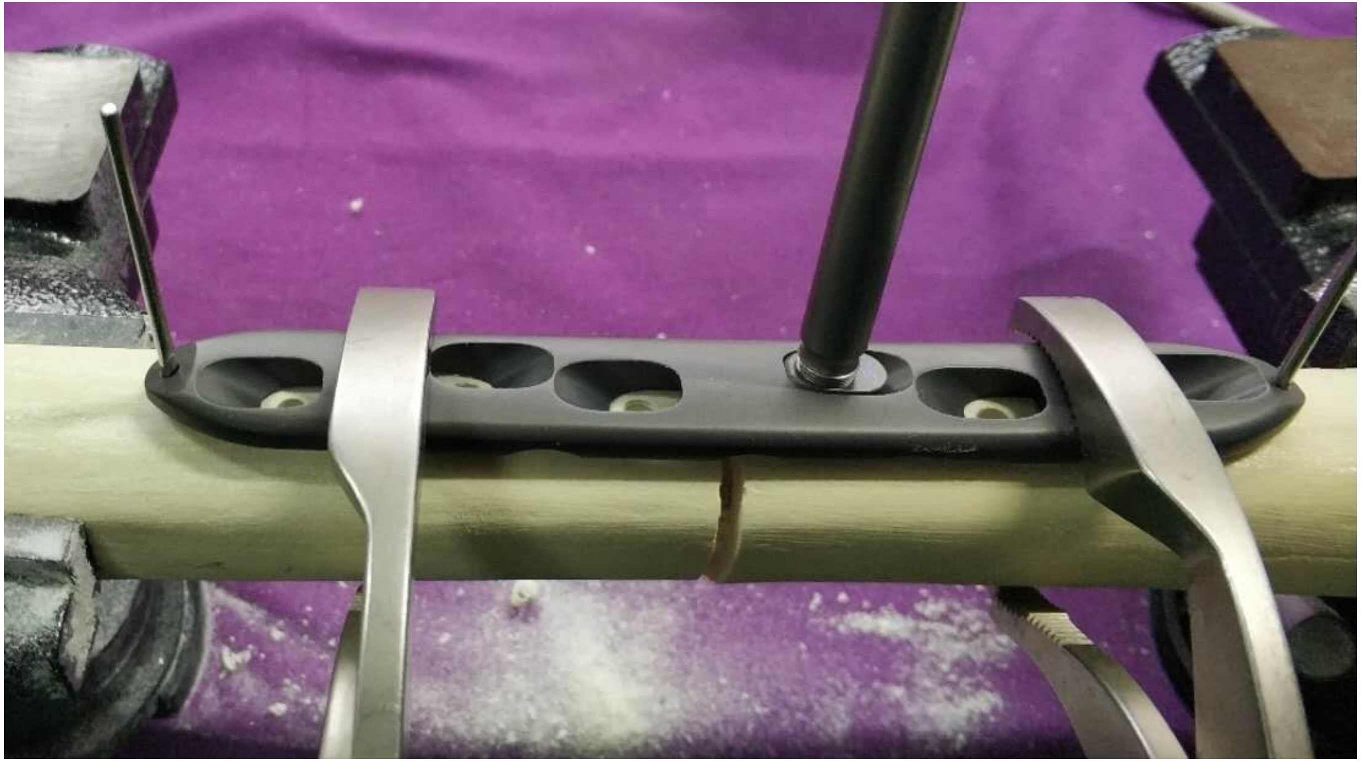

Step 9: fix the osteotomy with 4.5-mm locking screws

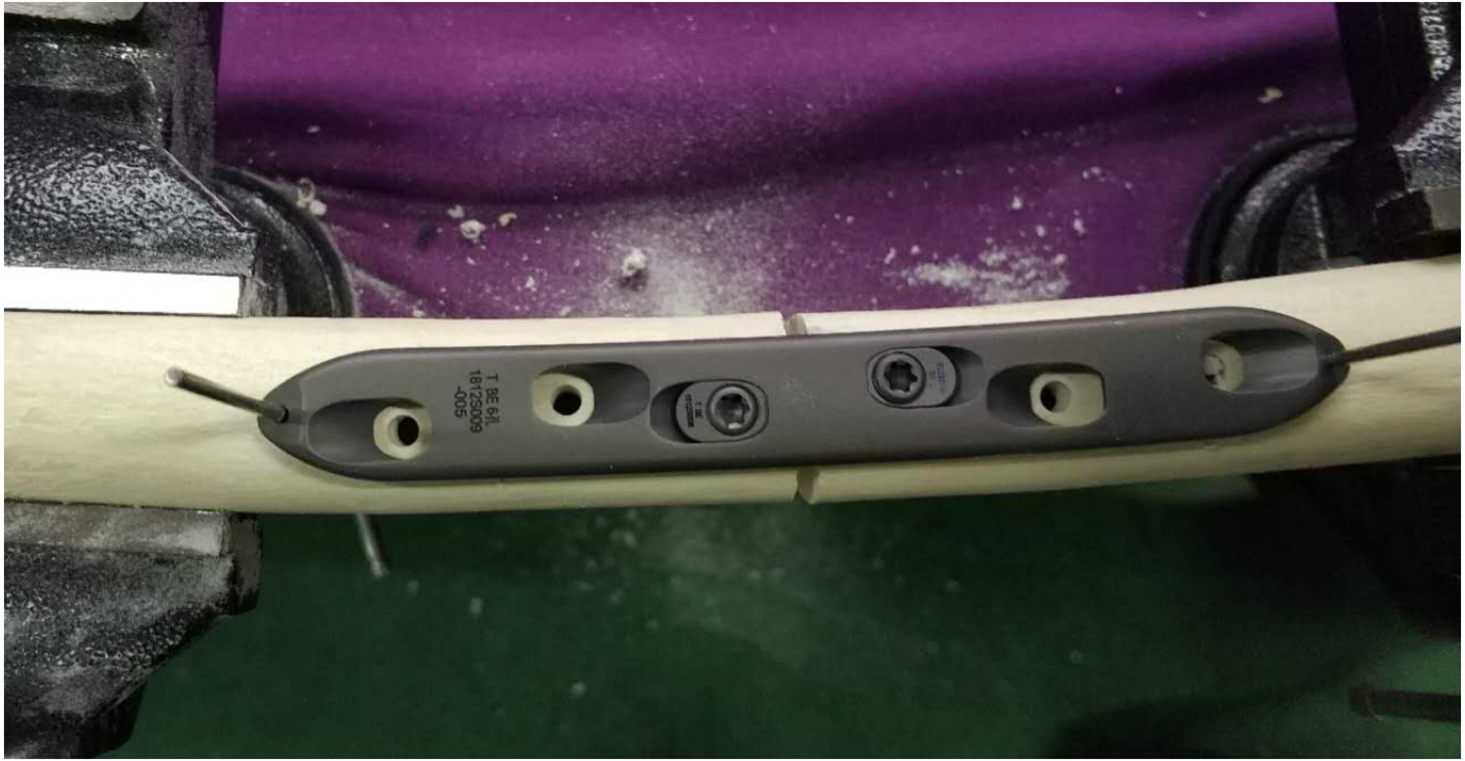

Step 10: place sliding element in other holes

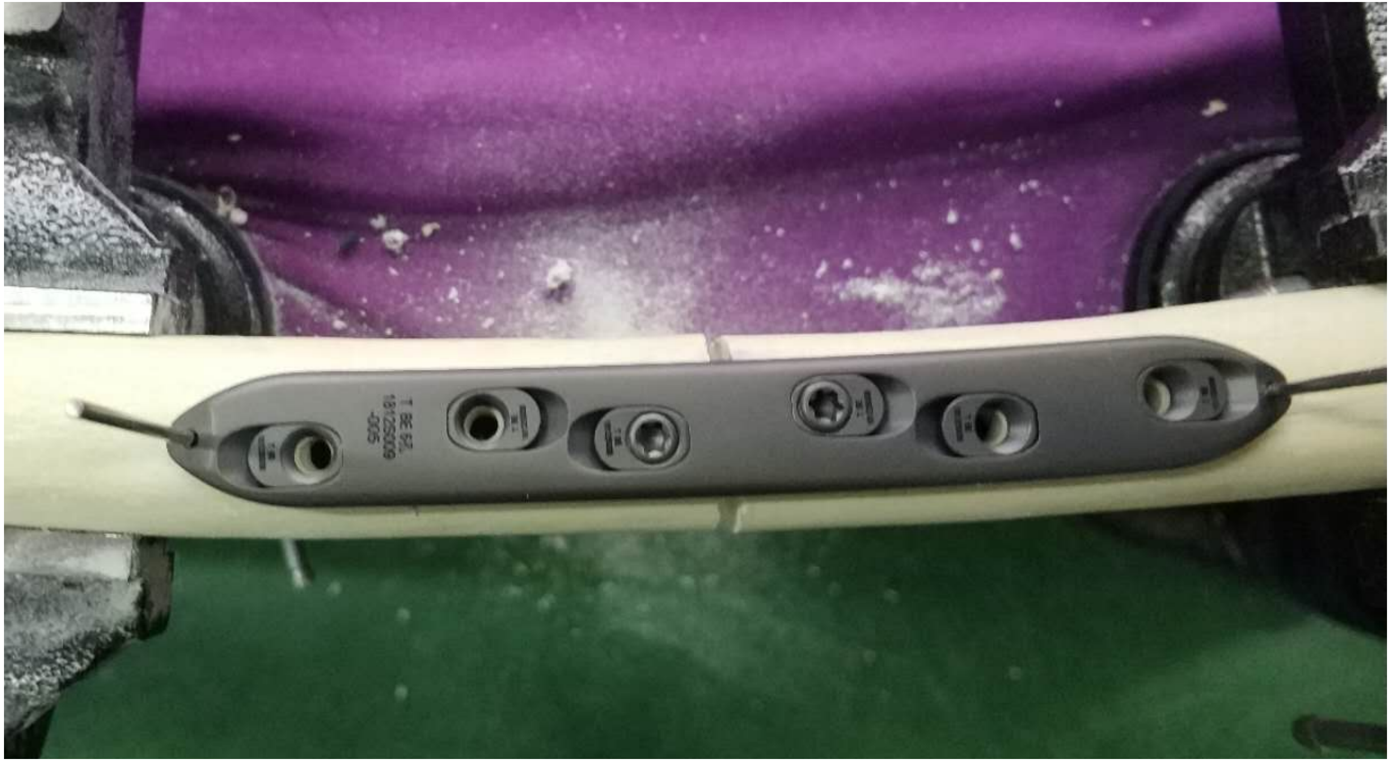

Step 11: complete the fixation

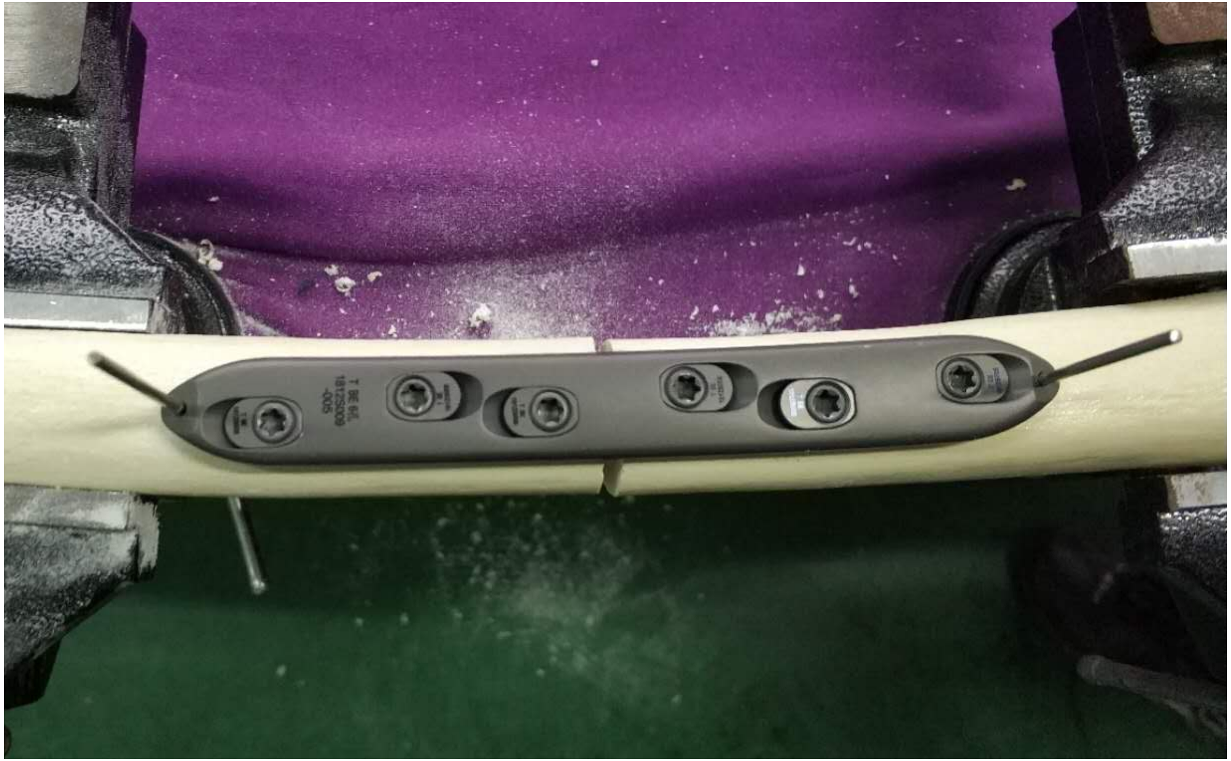

Step 12: remove the Kirsch wires

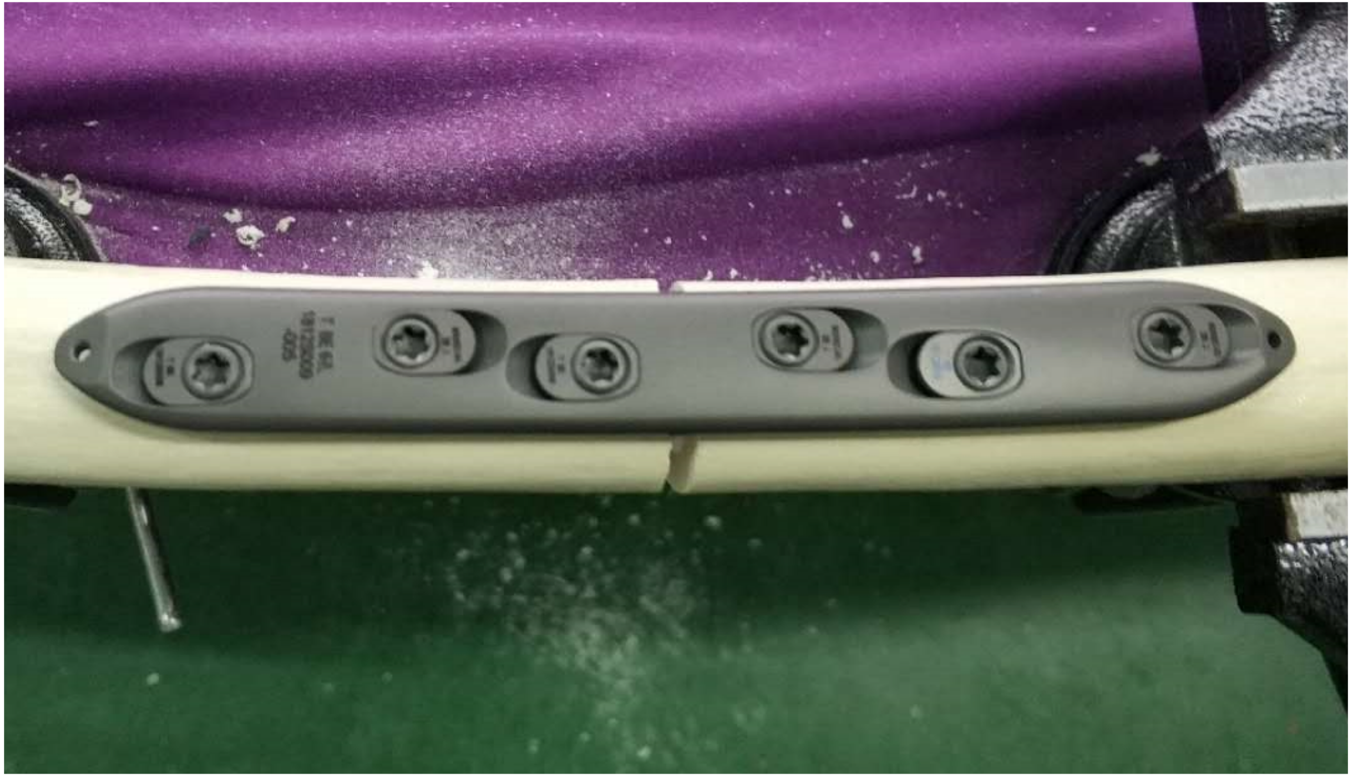

Supplement: Supplementary file 1 [file Data_Sheet_1.PDF]
