# Supplementary figures and images for: Axial Micromotion Locking Plate Construct Can Promote Faster and Stronger Bone Healing in an Ovine Osteotomy Model
Source: Front Bioeng Biotechnol. 2021 Jan 15;8:593448. doi: 10.3389/fbioe.2020.593448 (PMC7845656; doi:10.3389/fbioe.2020.593448)

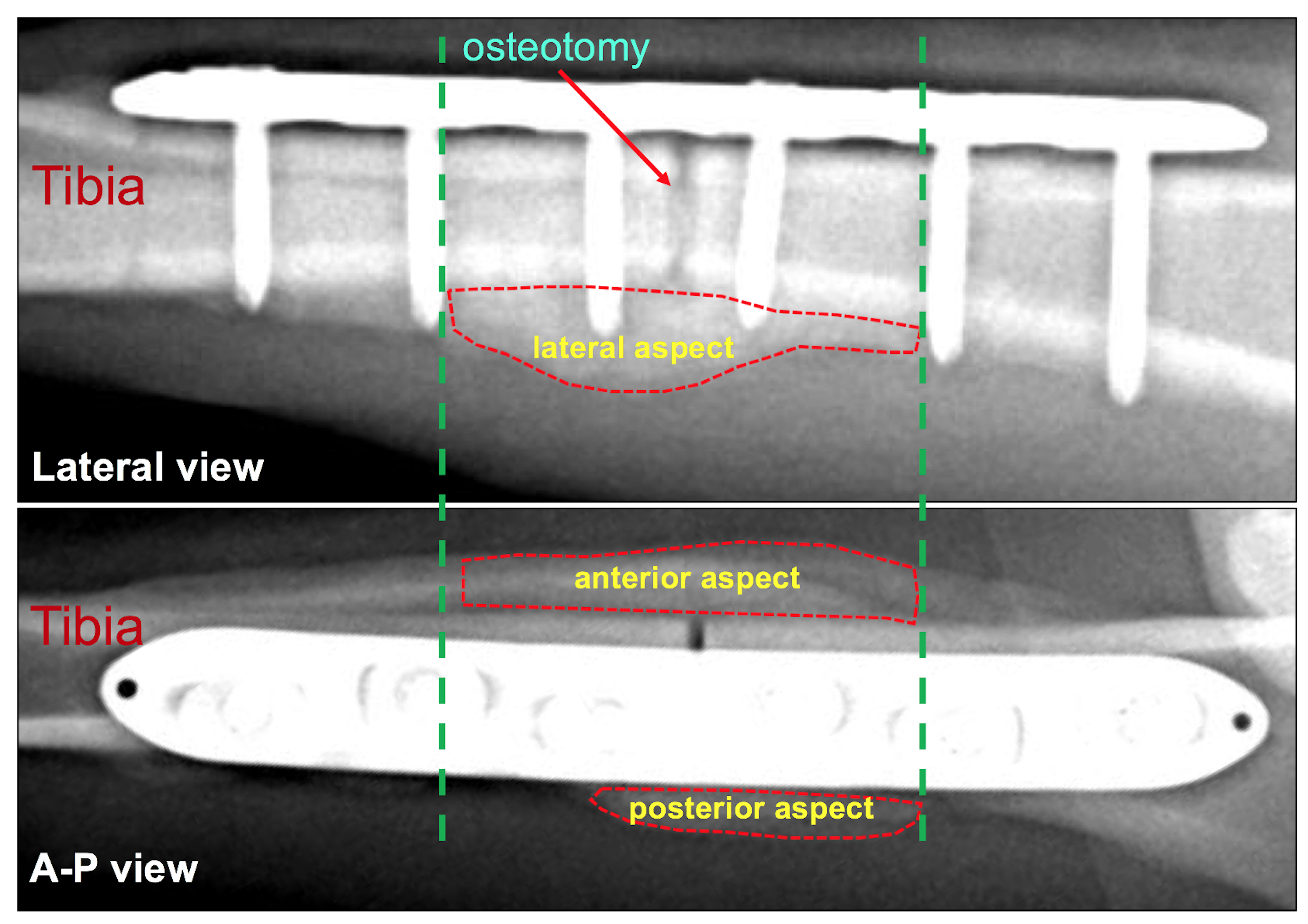

Supplement: Supplementary file 3 [file Image_1.JPEG]

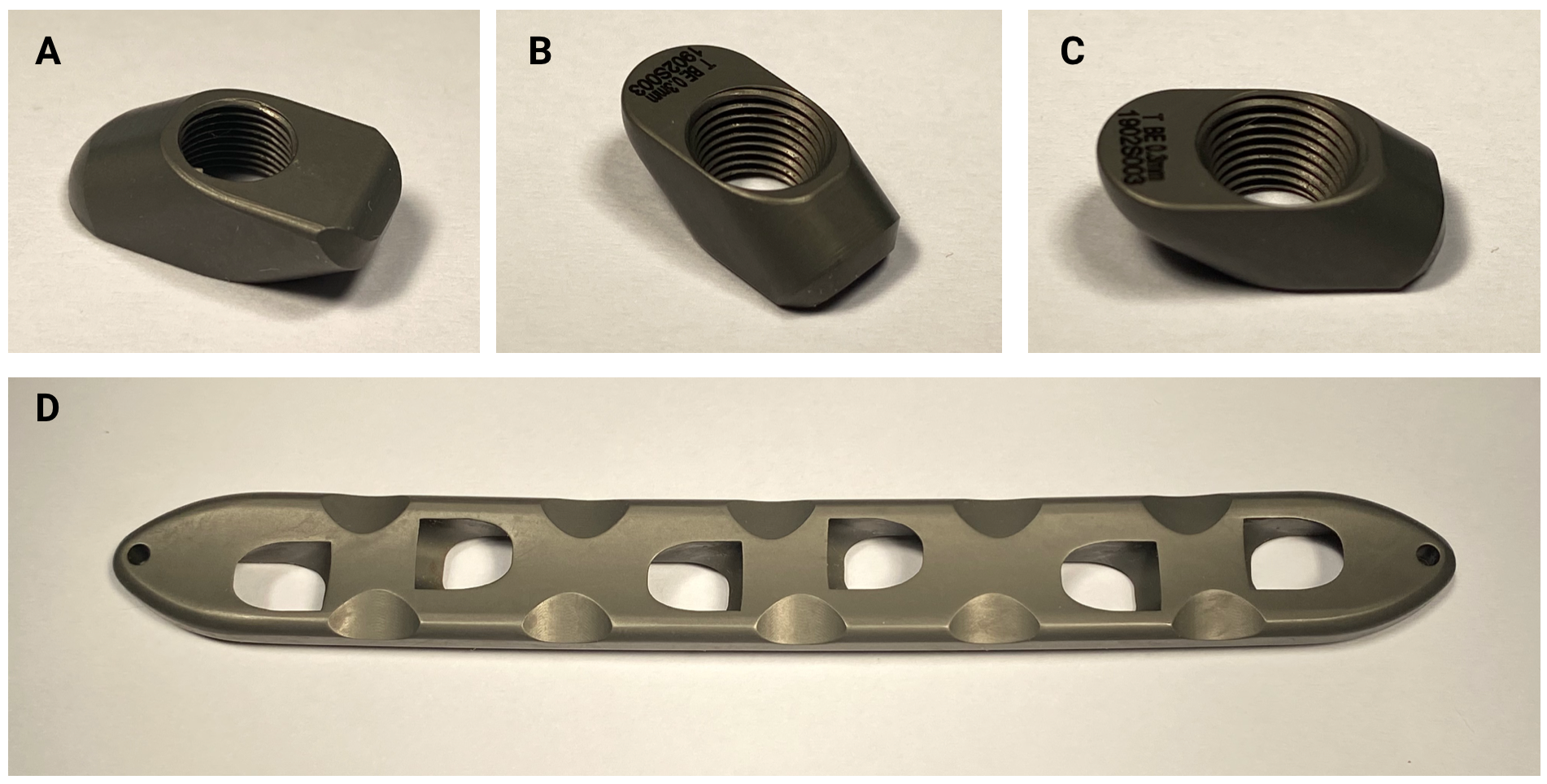

Supplement: Supplementary file 4 [file Image_2.PNG]
